# Unraveling the Pathogenesis of MDS: The NLRP3 Inflammasome and Pyroptosis Drive the MDS Phenotype

**DOI:** 10.3389/fonc.2016.00151

**Published:** 2016-06-16

**Authors:** David A. Sallman, Thomas Cluzeau, Ashley A. Basiorka, Alan List

**Affiliations:** ^1^Malignant Hematology Department, H. Lee Moffitt Cancer Center and Research Institute, Tampa, FL, USA; ^2^Hematology Department, Centre Hospitalier Universitaire of Nice, Nice, France; ^3^Faculty of Medicine, University Nice Sophia Antipolis, Nice, France; ^4^Mediterranean Center of Molecular Medicine, INSERM U1065, Nice, France; ^5^French Group of Myelodysplasia, Paris, France; ^6^Cancer Biology Ph.D. Program, H. Lee Moffitt Cancer Center and Research Institute, University of South Florida, Tampa, FL, USA

**Keywords:** MDS, pyroptosis, inflammasome, NLRP3, S100A9, TLR

## Abstract

Myelodysplastic syndromes (MDS) are characterized by bone marrow cytological dysplasia and ineffective hematopoiesis in the setting of recurrent somatic gene mutations and chromosomal abnormalities. The underlying pathogenic mechanisms that drive a common clinical phenotype from a diverse array of genetic abnormalities have only recently begun to emerge. Accumulating evidence has highlighted the integral role of the innate immune system in upregulating inflammatory cytokines *via* NF-κB activation in the pathogenesis of MDS. Recent investigations implicate activation of the NLRP3 inflammasome in hematopoietic stem/progenitor cells as a critical convergence signal in MDS with consequent clonal expansion and pyroptotic cell death though caspase-1 maturation. Specifically, the alarmin S100A9 and/or founder gene mutations trigger pyroptosis through the generation of reactive oxygen species leading to assembly and activation of the redox-sensitive NLRP3 inflammasome and β–catenin, assuring propagation of the MDS clone. More importantly, targeted inhibition of varied steps in this pathway restore effective hematopoiesis. Together, delineation of the role of pyroptosis in the clinical phenotype of MDS patients has identified novel therapeutic strategies that offer significant promise in the treatment of MDS.

## Introduction

Myelodysplastic syndromes (MDS) represent a heterogeneous group of clonal hematopoietic neoplasms hallmarked by ineffective erythropoiesis, bone marrow (BM) dysplasia, and risk of transformation to acute myeloid leukemia (AML). In depth molecular characterization has shed significant insight into the molecular architecture of this disease that impacts diagnosis and prognosis, while illustrating the genetic complexity of these malignancies. Specifically, the molecular heterogeneity in MDS involves mutations of splicing gene machinery, epigenetic regulation, differentiation, and cell signaling. How these seemingly diverse perturbations result in a common clinical and hematological phenotype remains unexplained. Recent investigations have identified a critical role of innate immune and inflammatory signaling in the development of MDS, as well as provide a link between the genetic heterogeneity and the myelodysplastic phenotype. More importantly, deciphering the key biological features of this disease offers the opportunity for novel, biologically rational therapeutic strategies to target the underlying disease pathogenesis.

## TLR Signaling and Role in MDS

**Figure 1:**
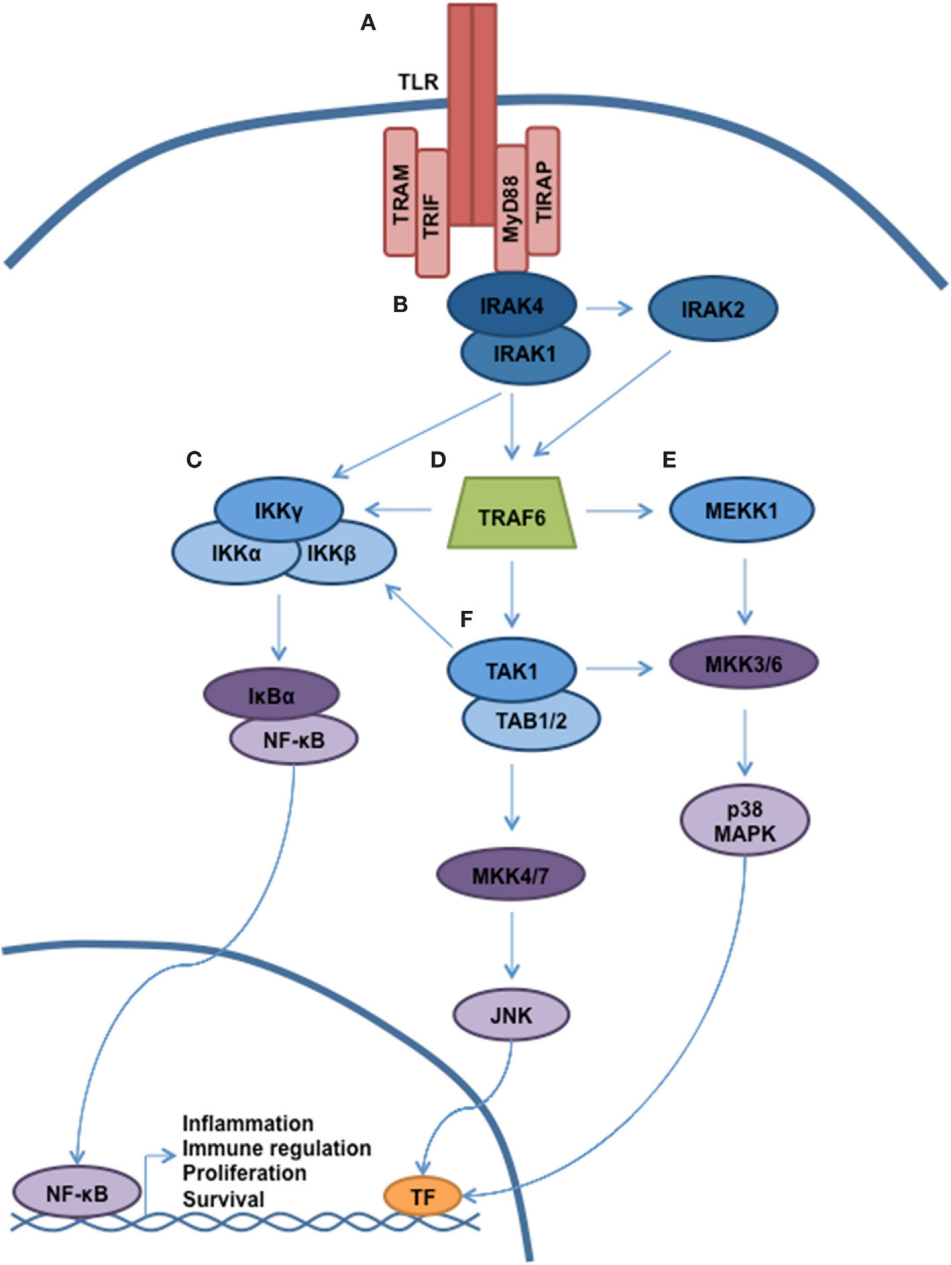
**TLR signaling governs multiple cellular processes through a complex signaling network**. A simplified schematic of TLR signaling is presented. **(A)** The majority of the TLRs reside in the plasma membrane (with the exception of TLR3, TLR7–9). Following receptor ligation, a number of signaling adaptor proteins will be recruited, including MyD88, TIRAP, TRAM, and TRIF. Depending on the particular stimulus, certain TLRs will become activated, resulting in recruitment of specific adaptor proteins, kinases, and ubiquitin ligases that will help propagate signaling. **(B)** Through interaction with MyD88, the serine/threonine kinase IRAK4 will be recruited. Subsequently, IRAK1 and IRAK2 will be activated. **(C)** Autoubiquitination of TRAF6 allows for recruitment of the IKK complex (IKKα, IKKβ, and IKKγ/NEMO). Phosphorylation of IKKβ by TAK1 allows for phosphorylation of NF-κB, thereby targeting Ikβ proteins for degradation. Liberated NF-κB can enter into the nucleus and direct transcription. **(D)** MyD88 and IRAK join to form a large multiprotein complex including the E3 ubiquitin ligase TRAF6. **(E,F)** Activation of TAK1 and MEKK1 result in a signaling cascade, leading to the activation of MAPK signaling and modulation of gene expression.

Antigen recognition by the innate immune system is accomplished *via* the interaction of pathogen-associated molecular patterns (PAMPs) with pattern-recognition receptors (PRRs). Toll-like receptors (TLRs) represent the most important members of the PRR family, and activation of TLRs triggers multiple cellular processes *via* a complex signaling cascade, as shown in Figure 1 (1, 2). In humans, 10 TLRs (TLR 1–10) have been identified, and each TLR is composed of a leucine-rich ectodomain, a transmembrane domain, and a toll-interleukin 1 receptor (TIR) domain. The TIR domain is responsible for signal transduction, which occurs through the recruitment of specific adaptor molecules, including myeloid differentiation primary response 88 (MyD88), TIR-domain-containing adaptor protein (TIRAP), TIR-domain-containing adapter-inducing interferon-β (TRIF), and the TRIF-related adaptor molecule (TRAM) (3). MyD88 is utilized by all TLRs with the exception of TLR3, which employs TRIF. Together, TLR signaling occurs either *via* MyD88-dependent pathways leading to the activation of nuclear factor kappa-light-chain-enhancer of activated B cells (NF-κB) and mitogen-activated protein kinases (MAPKs) with induction of inflammatory cytokines, or through TRIF-dependent induction of type 1 interferon (IFN) through the activation of IFN regulatory factor 3 (IRF3) (2). TLR4 is activated by an array of ligands, including lipopolysaccharide (LPS), which triggers both MyD88 and TRIF-dependent signaling through interaction with the specific adaptor molecules TIRAP and TRAM, respectively. Of interest, TLR4-mediated induction of inflammatory cytokines requires activation of both pathways, which is distinct from all other TLRs (4, 5). MyD88-dependent activation of NF-κB and additional effectors occurs through the sequential activation of IL-1 receptor-associated kinases (IRAKs). Specifically, ligand-induced dimerization of TLR results in IRAK4 recruitment and activation followed by the activation of IRAK1 and IRAK2 (6). IRAK then dissociates from the receptor complex to create a large multiprotein complex involving the E3 ubiquitin ligase TNF receptor-associated factor-6 (TRAF6), the mitogen-activated kinase kinase kinase family member, TGF-β-activated kinase (TAK)-1, IκB kinase (IKK), TAB2/TAB3, and NEMO, respectively (7, 8). TAK1 phosphorylation of IKK-β triggers the phosphorylation of NF-kB-bound IκB proteins, targeting them for ubiquitin-dependent degradation and liberating NF-κB dimers to enter the nucleus.

Hematopoietic cytokines play an indispensable role in the maturation of myeloid precursor cells under steady state conditions and particularly during stress, such as infection and inflammation (9). Importantly, these cytokines have pleiotropic effects on hematopoietic cells, including regulation of proliferation, maturation, and activation. More recent investigations have shown that hematopoietic stem and progenitor cells (HSPCs) also harbor TLRs, including TLR4, which upon activation by LPS lead to HSPC proliferation. As HSPCs do not have intrinsic effector function, TLR activation plays a distinct role of localization to sites of infection/inflammation (e.g., lymph nodes) and the replenishment of effector cells (10, 11). Although previous understanding of HSPCs reasoned that their quiescent state resulted in protection from mutagenesis, recent work has highlighted that HSPCs have significant dependence on non-homologous end-joining DNA repair, and this process increases susceptibility to somatic mutations (12). In addition, innate immune-induced changes in the surrounding stroma, i.e., the mesenchymal cells, could potentially foster propagation of these clones, which would otherwise not have had a proliferative advantage (13, 14).

**Figure 2:**
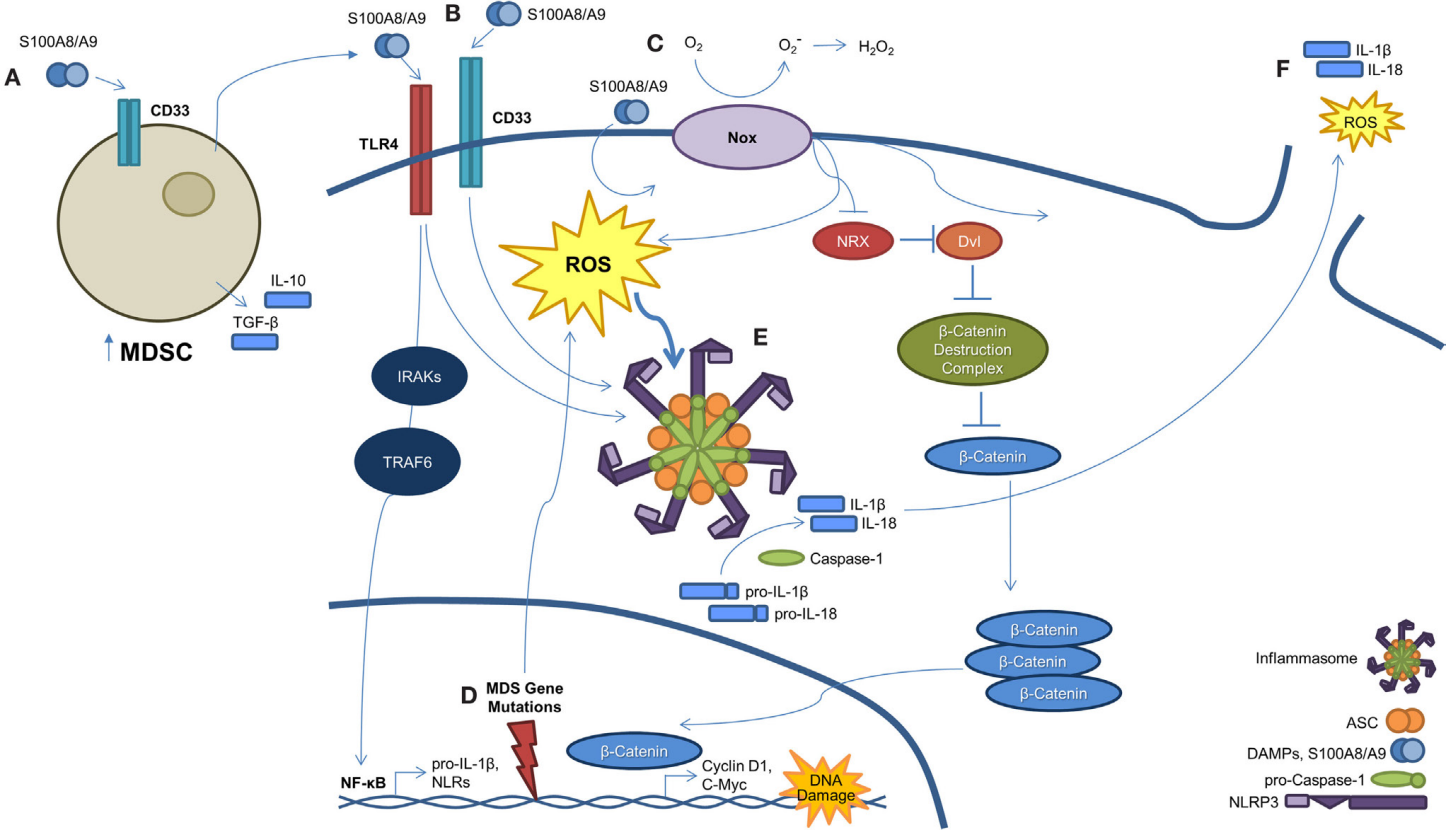
**A S100A9-pyroptosis circuit provokes phenotypes manifest in MDS**. **(A)** Myeloid-derived suppressor cells (MDSCs) are markedly expanded in the BM of MDS patients. MDSCs produce and secrete S100A9, which functions to mediate progenitor cell death and also activates MDSCs in an autocrine manner. **(B)** S100A8/A9 binds both CD33 and TLR4, resulting in NLRP3 inflammasome assembly. Ligation of S100A8/A9 to TLR4 through the IRAK–TRAF6 axis results in NF-κB-mediated transcription and subsequent production of pro-inflammatory cytokines, like pro-IL-1β and -IL-18, along with inflammasome components. **(C)** S100A8/A9 promotes activation of NAPDH oxidase (NOX), which results in a dual function. First, NOX proteins generate ROS, which serve to active NLRs and trigger inflammasome assembly. Second, NOX-derived ROS result in the oxidation of nucleoredoxin (NRX), leading to its dissociation from disheveled (Dvl). Once dissociated, Dvl suppresses the β-catenin destruction complex, resulting in stabilization of β-catenin. This allows β-catenin to enter the nucleus and induce transcript of TCF/LEF-controlled genes, including *cyclin-D1* and *c-Myc*, which are essential to self-renewal. **(D)** MDS-related gene mutations activate NF-κB and NLRP3 *via* NOX-generated ROS. **(E)** Formation of the NLRP3 inflammasome complex occurs as a consequence of ROS activation and DAMP signaling. Once activated, inflammasomes mediate conversion of pro-caspase-1 to its mature and catalytically active form. Active caspase-1 cleaves pro-IL-1β and pro-IL-18 to their mature forms. **(F)** Pyroptosis ensues with loss of membrane integrity resulting in the release of pro-inflammatory cytokines, ROS, and other intracellular contents into the extracellular milieu.

More recently, multiple components of the TLR signaling cascade have been associated with MDS (Figure 2). Specifically, TLR4 is upregulated in HSPCs of MDS patients and directly correlates with annexin-V positivity in BM mononuclear cells (BM-MNC) and CD34+ cells (15). In MDS patients, particularly in MDS with deletion of 5q (del(5q)), TRAF6 is overexpressed owing to haploinsufficiency of microRNA-145 and microRNA-146a, negative regulators of TRAF6 (16). In addition, TRAF6 is also important in cell survival signaling of MDS HSPCs. IRAK1, the adaptor kinase activated upon TLR4 stimulation, is overexpressed in MDS progenitors (17, 18). Of therapeutic importance, IRAK 1/4 inhibition and/or RNA interference (RNAi) suppression of IRAK1 was selectively cytotoxic to MDS HSPCs without consequence to normal CD34+ cells (18). As IRAK inhibitor therapy was shown to induce BCL-2 expression, suggesting a potential resistance mechanism, Rhyasen and colleagues provide initial evidence to support combination therapy of an IRAK inhibitor with a BCL-2 antagonist in both *in vitro* and *in vivo* models of MDS.

## Inflammatory Mediatiors as a Driver of MDS Pathogenesis through Modulation of the BM Microenvironment

In addition to PAMPs, damage-associated molecular patterns (DAMPs) or alarmins are molecules that are released during inflammation or cell death, which can modulate the innate immune system through TLR signaling, particularly TLR4 (19). Specifically, more recent work has highlighted that S100A8 and S100A9 are endogenous ligands of TLR4 and primarily exist as a heterodimeric complex, calprotectin, leading to the induction of an inflammatory response through autocrine and paracrine mechanisms (20). Previously, S100A8/A9 levels have been found to not only be elevated in inflammatory diseases but also play a unique role in the underlying pathogenesis of these diseases (20, 21). S100A8/A9 has been implicated in tumor promotion including colon cancer cell proliferation, where it also provides a plausible mechanism for colitis-associated colon cancer initiation and progression (22). How this inflammatory mechanism is linked to innate immunity and cancer progression was unclear until recent investigations. Specifically, Cheng and colleagues demonstrated *in vivo* that this complex is of paramount importance for tumor progression through the induction of myeloid-derived suppressor cells (MDSCs), which lead to the suppression of antitumor immunity (23). Of interest, MDSCs not only respond to but also produce S100A8/S100A9, leading to autocrine stimulation that is extinguishable by antibody neutralization (24).

Although a pro-inflammatory state leading to chronic immune stimulation and hematopoietic senescence has been linked to MDS (25), the underlying mechanisms relating to this pathogenic process have only been recently advanced. Of note, we have demonstrated that polyclonal MDSCs (CD33+/Lin−/HLA−DR−) are significantly expanded in the BM of MDS patients. As shown in Figure 2, this expansion is mediated through S100A9 ligand activation of CD33, leading to production of the suppressive cytokines interleukin-10 (IL-10) and transforming growth factor-β (TGF-β) (26). This immune suppressive mechanism occurs through the immunoreceptor tyrosine-based inhibition motif (ITIM) of CD33. Furthermore, S100A9 transgenic mice manifest a phenotype similar to MDS patients with age-dependent dysplastic changes and impaired hematopoiesis. Disruption of the S100A9/CD33/TLR4 circuit was able to restore effective hematopoiesis. Together, these data highlight that the BM microenvironment, and in particular MDSCs, are integrally linked to the pathogenesis of MDS. Although MDSC expansion has been found to occur in relation to senescence-dependent changes in the BM (27), this expansion alone is inadequate to induce an MDS phenotype and suggests that the upregulation of pro-inflammatory molecules, such as S100A9, could be the initiating event in the development of MDS. Additional evidence supporting this hypothesis is that the MDSCs derived from MDS patients are genetically distinct from the MDS clone and lack the corresponding somatic gene mutations and chromosomal abnormalities (26). As proof of this concept, recent elegant work has shown in deletion 5q MDS that ribosomal haploinsufficiency of *Rps14* induces S100A8/A9, which is sufficient to block erythroid differentiation in normal erythroid cells, while inactivation restores effective erythropoiesis in haplodeficient cells (28).

## NLRP3 Inflammasome Activation and Induction of Pyroptosis Underly the MDS Phenotype

Although the above studies clearly characterize the importance of inflammation and the innate immune system in the underlying pathogenesis of MDS, the precise mechanism of cell death was poorly understood. Although apoptosis and autophagy may play a role in MDS (see below), more recent work has demonstrated pyroptosis, a novel caspase-1-dependent pro-inflammatory cell death induced by DAMP activation of PRPs (29–31), to be the fundamental driver of HSPC death (Figure 2). Specifically, pyroptosis is executed through inflammasome formation, cytosolic, multiprotein complexes composed of nucleotide-binding domain and leucine-rich repeat pattern-recognition receptors (NLRs). NLR family pyrin domain-containing 3 (NLRP3) is the best characterized NLR and is activated in response to multiple DAMPs, leading to the recruitment of an apoptosis-associated speck-like protein containing a caspase-recruitment domain (ASC). Following recruitment to NLRP3, ASC binds pro-caspase-1 leading to its cleavage and activation (32). Caspase-1 activation results in multiple cellular processes, including nuclear condensation, conversion of precursors of the inflammatory cytokines interleukin 1β (IL-1β) and IL-18 to their active form, and pore formation leading to the influx of cations with cell swelling and osmotic lysis (32). Masters and colleagues provided an initial link of pyroptosis to hematopoiesis by demonstrating that inflammatory-mediated NLRP1a inflammasome activation by endotoxin resulted in pyroptosis of HSPCs and consequent cytopenias (30). In addition, intracellular S100A8/A9 heterodimers form a scaffold for the membrane insertion and activation of NADPH oxidase to generate reactive oxygen species (ROS), which reciprocally induce NF-κB activation and inflammasome assembly leading to generation of pro-inflammatory cytokines (33). These authors show that, in particular, S100A9 is critical for NF-κB-mediated transcription of inflammasome components or “inflammasome priming” and is also a more potent inducer of cytokines than S100A8. Of importance, S100A9-induced lytic cell death provides a feed-forward amplification loop, whereby additional S100A9 is released along with other DAMPs, such as the TLR4 ligand high mobility group box-1 protein (HMGB1) and inflammasome components, including ASC (34, 35).

How an inflammatory mediated cell death could be integrated into a clonally heterogeneous-driven process had remained elusive (Figure 2). To this end, we recently described a common pathway licensed by somatic mutations and/or DAMP signals to induce inflammasome activation and pyroptosis, resulting in the phenotypic features of MDS (36). Comparison of MDS BM-MNC to normal controls illustrated that pyroptosis, but not apoptosis, was profoundly primed as evidenced by marked upregulation of caspase-1 (~209-fold) and NLRP3 (~48-fold) without any difference in caspase-3 expression. In addition, fluorescent microscopy showed co-localization of caspase-1 with NLRP3, confirming inflammasome assembly irrespective of the international prognostic scoring system (IPSS) risk category. Pyroptosis execution, defined as active caspase-1^+^/active caspase-3^+^/annexin-V^+^ cells, was significantly increased in MDS HSPCs and their progeny compared to normal controls. Again, the percentage of pyroptotic cells was the predominant cause of cell death in comparison to apoptosis (active caspase-3/7^+^/active caspase-1^−^/annexin-V^+^). In addition, BM plasma levels of S100A9 were significantly increased in MDS patients. Furthermore, treatment of normal BM-MNC with recombinant human S100A9 (rhS100A9) recapitulated the above MDS phenotype with activation of pyroptosis and induction of NLRP3 inflammasome formation, which was also observed in the S100A9 transgenic mouse model. Of particular importance, myelodysplastic HSPCs appear inherently primed for pyroptotic response to alarmins, such as S100A9, compared to their normal counterparts by virtue of overexpression of TLRs and downregulation of the primary negative regulator of inflammasome activity, the pyrin domain-only protein (POP)-1. Such TLR upregulation occurs in the setting of chronic and sustained TLR activation, driving myeloid hematopoietic skewing, loss of HSC quiescence, and telomere erosion (37, 38). Notably, pyroptotic cell death is characterized by the activation of plasma membrane cation channels, which trigger mitochondrial depolarization and cell swelling (39). MDS BM-MNC demonstrate unexplained macrocytosis. In comparison to normal BM-MNC, we show that MDS specimens display rapid and sustained uptake of the cationic dye ethidium bromide, confirming cation channel activation in accordance with pyroptotic and MDS hallmarks. Transient receptor potential melastatin-2 (TRPM2), a cation channel found on hematopoietic cells, directs calcium influx and corresponding increases in cell volume following activation (40). As TRPM2 has been linked to NLRP3 activation (41), TRPM2 may mediate the pyroptotic-driven cell swelling observed in MDS. More importantly, delineating the above processes has allowed for the development of novel therapeutic strategies. As an example, using a CD33/IgG chimera to neutralize S100A9 reduced the pyroptotic fraction and improved *in vitro* colony-forming capacity in primary MDS patient specimens. In our S100A9 transgenic mouse model, inflammasome inhibition using ICTA, an icariin derivative that inhibits NLRP3 inflammasome activation, was able to suppress pyroptosis and β-catenin activation, with corresponding restoration of effective hematopoiesis.

Previous studies have demonstrated that the superoxide-generating NADPH oxidase (Nox) induces ROS production leading to inactivation of nucleoredoxin (NRX) in the β-catenin destruction complex, to activate the Wnt/β–catenin pathway (42). We investigated whether this pathway could be activated by S100A9 and/or genetic mutations to promote clonal expansion (36). Indeed, not only was ROS elevated and β-catenin markedly activated in MDS patient specimens compared to normal controls, but also the treatment of normal BM-MNC with rhS100A9 was able to recapitulate these findings. Activation of this ROS/β–catenin pathway along with inflammasome activation was induced by spliceosome mutations both *in vitro* and in *in vivo* murine models. Abrogation of this pathway by treatment with the antioxidant *N*-acetylcysteine (NAC), or specifically with the NAPDH oxidase (NOX) inhibitor diphenylene iodonium (DPI), prevented ROS production and β-catenin activation (i.e., nuclear β-catenin). Additionally, these findings were not specific to spliceosome mutations but were also observed in murine models driven by mutations of the epigenetic regulatory genes, including *TET2* and *ASXL1*, indicating a common point of convergence by functionally diverse somatic gene mutations in MDS.

## Additional Cell Death Mechanisms in MDS

**Figure 3:**
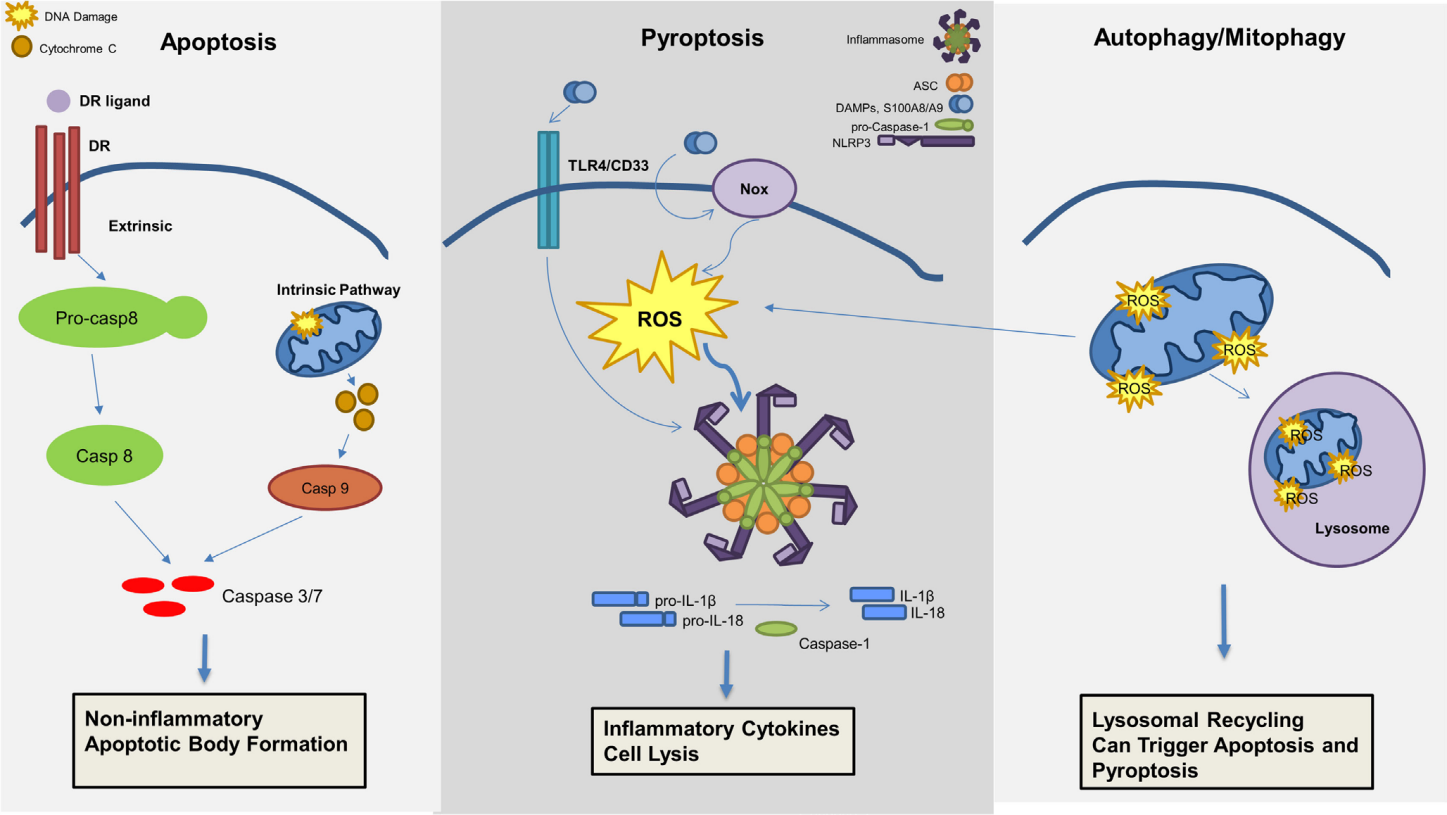
**Mechanisms of cell death involved in the pathogenesis of myelodysplastic syndromes (MDS)**. Apoptosis, pyroptosis, and autophagy all contribute to cell death in MDS. Apoptosis, a non-inflammatory cell death, can be triggered through an extrinsic or intrinsic pathway leading to effector caspase activation and apoptotic body formation. Pyroptosis is an inflammatory cell death mechanism, which is triggered by damage-associated molecular patterns (DAMPs), in particular S100A8/S100A9, leading to ROS production and inflammasome activation resulting in production of pro-inflammatory cytokines (i.e., IL-1β and IL-18) and caspase-1 activation with consequent cell lysis. Lastly, mitophagy, or the selective degradation of mitochondria through lysosomal recycling and an autophagic mechanism, leads to ROS production and can induce both apoptosis and pyroptosis.

Although recent investigations have highlighted the critical role of pyroptosis in the pathogenesis of MDS as described above, additional cell death mechanisms may also contribute to the MDS phenotype (Figure 3). Apoptosis is well described and can be distinguished by two initiation pathways, the intrinsic and extrinsic mitochondrial pathways, which both converge on caspase-3 activation as the principal cell death executioner. The extrinsic pathway is dependent upon cell death receptors and its ligands, including tumor necrosis factor alpha (TNFα)/TNF receptor (TNF-R), Fas/Fas ligand, or TNF-related apoptosis-inducing ligand (TRAIL)/TRAIL receptor (TRAIL-R) (43). Ligand activation of receptor will activate a signaling cascade that is also dependent on caspase-8 activation (44). In contrast, the intrinsic pathway is dependent on mitochondria and BCL-2 members, inducing caspase-9 activation (45).

Several studies have shown increased apoptosis in MDS BM-MNC (46, 47); however, these studies preceded recognition of pyroptosis and did not investigate caspase-1 activity. The specific apoptotic pathway leading to cell death in MDS patients appeared related to the stage of the disease, with activation of the extrinsic pathway predominantly in early-stage MDS versus intrinsic pathway activation during evolution of the disease. In early stages, an increase of apoptosis was attributed to an upregulation of TNFα, Fas/Fas ligand, and TRAIL (48–50). All of these ligands belong to the extrinsic pathway of apoptosis and were linked to ineffective hematopoiesis and peripheral blood cytopenias. Moreover, inhibition of the extrinsic pathway restored effective hematopoiesis (51). During MDS progression, activation of the intrinsic pathway of apoptosis occurs, implicating BCL-2 members. Whereas upregulation of proapoptotic proteins like Bak and Bax are demonstrable in lower-risk MDS, an increase in antiapoptotic proteins, including Bcl-2, Bcl-X_L_, and the Flice inhibitory protein (FLIP), was observed in high-risk MDS during progression to AML, due in part to NF-κB inhibition of apoptosis and cell proliferation (52, 53).

Autophagy is the catabolic process by which long-lived, superfluous, or damaged macromolecules and organelles are degraded by lysosomal hydrolases for recycling, thereby permitting cells to survive starvation and stressful conditions (54, 55). The autophagy process, from phagosome formation to lysosome fusion, involves approximately 30 autophagy-related proteins (56). Both pharmacological and genetic evidence indicate that autophagy plays pleiotropic functions in hematopoietic cell homeostasis and leukemogenesis. Autophagy exerts either pro-survival or tumor-suppressive functions, depending on both the context and the nature of the hematopoietic malignancy and initiating signal (57). Moreover, several autophagy genes, such as Beclin 1 and UV radiation resistance-associated gene (UVRAG), are now known to act as tumor suppressor genes (58). Given the cross talk between the autophagic and apoptotic pathways, autophagy has been implicated in cancer. Moreover, prolonged stimulation of autophagy can itself induce apoptosis (59). Autophagy also has a protective role through controlling oxidative stress as well as the accumulation of DNA damaging waste (57, 60). The selective removal of damaged mitochondria by autophagy, called mitophagy, has been well studied in MDS (61, 62). Increased caspase-dependent apoptosis, ROS, and mitochondrial damage are demonstrable in MDS (63). In erythroid precursors from lower-risk MDS patients, mitochondria sequestration in the autophagosome is evident, indicating intrinsic differentiation defects and higher levels of dysfunctional, iron-saturated mitochondria (64). This mitophagy defect generates ROS that contribute to DNA damage. A possible explanation may be that a basal level of autophagy in MDS occurs in response to oxidative stress to inhibit apoptosis induction. Alternatively, TLR4 activation alone can trigger autophagy by MyD99- and TRIF-induced dissociation of Beclin 1 from its inhibitor Bcl-2 (65). To date, no mutations have been identified in autophagic machinery in MDS (17, 66). However, cytogenetic abnormalities localized in genes belonging to the autophagy network were identified in AML (67). Taken together, these data suggest a possible role for autophagy in MDS, and thus in transformation of MDS to AML. Autophagy and mitophagy could be beneficial to primary oncogene-mediated leukemic transformation and for the survival of neoplastic progenitors in a hostile milieu (67).

## Conclusion

Although the association of inflammation and chronic immune stimulation has long been associated with MDS, understanding the specific role in the underlying pathogenesis has only recently been recognized. TLR signaling and alterations in the BM microenvironment, particularly MDSCs and their autocrine alarmin S100A9, sustain an inflammatory milieu that propagate the MDS clone. The pro-inflammatory environment coupled with genetic mutations and/or DAMP signals, activate inflammasomes and the induction of HSPC proliferation and pyroptosis within the MDS clone. More importantly, deciphering the key biological features of MDS has unveiled multiple potential strategies for future therapeutic intervention to improve outcomes for MDS patients.

## Author Contributions

DS and AB wrote the paper, created the figures, and gave final approval. TC and AL wrote the paper and gave final approval.

## Conflict of Interest Statement

The authors declare that the research was conducted in the absence of any commercial or financial relationships that could be construed as a potential conflict of interest.
